# Supplementary material for: Induction of Multiple Alternative Mitogenic Signaling Pathways Accompanies the Emergence of Drug-Tolerant Cancer Cells
Source: Cancers (Basel). 2024 Feb 29;16(5):1001. doi: 10.3390/cancers16051001 (PMC10930612; doi:10.3390/cancers16051001)
Supplement: Supplementary file 1 [file cancers-16-01001-s001.zip › cancers-2874917-supplementary.pdf]

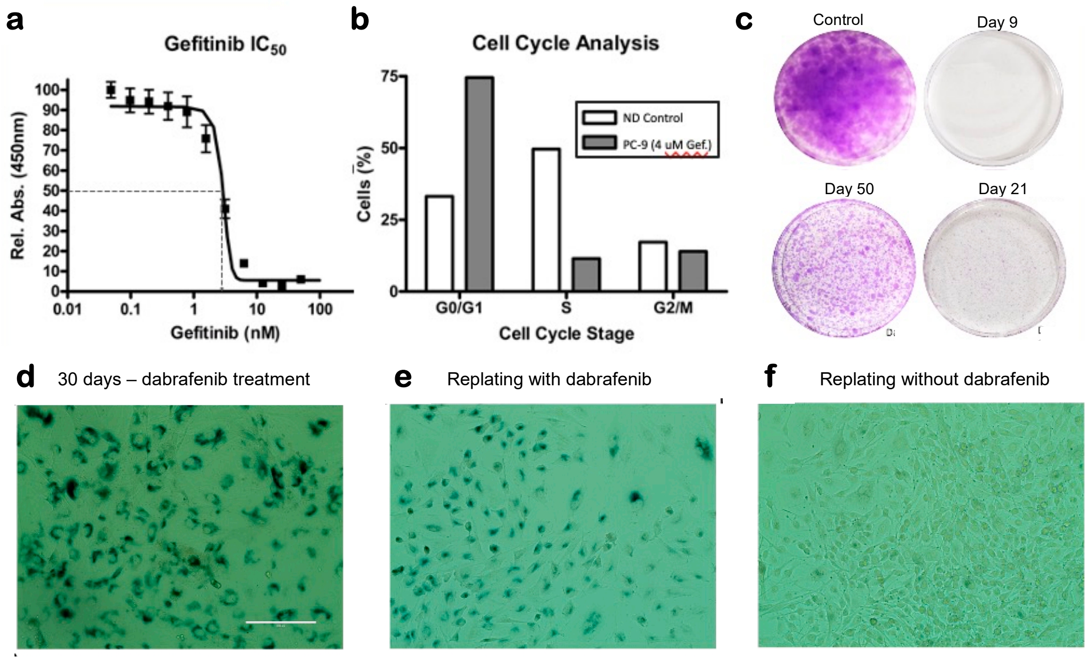

Figure S1. Development of drug-tolerant features in PC9 and SKMEL28 cells.. **a)** Dose-response of the effect of gefitinib on PC9 cells (3 day growth assay). **b)** Cell cycle stage of PC9 cells measured by propidium iodine staining and flow cytometry after 8 days growth either with or without gefitinib **c**) Clonal growth assay for 100,000 PC9 cells plated onto 100 mm dishes either without (control) or with gefitinib. At the indicated day, cells were fixed with methanol and stained with crystal violet. **d**) β-galactosidase staining of SKMEL28 cells cultured for 30 days in the presence of dabrafenib. **e**) the same cells following trypsinization and replating in the presence of dabrafenib for 3 days **f**) cells trypsinized and replated in the absence of dabrafenib for 3 days.

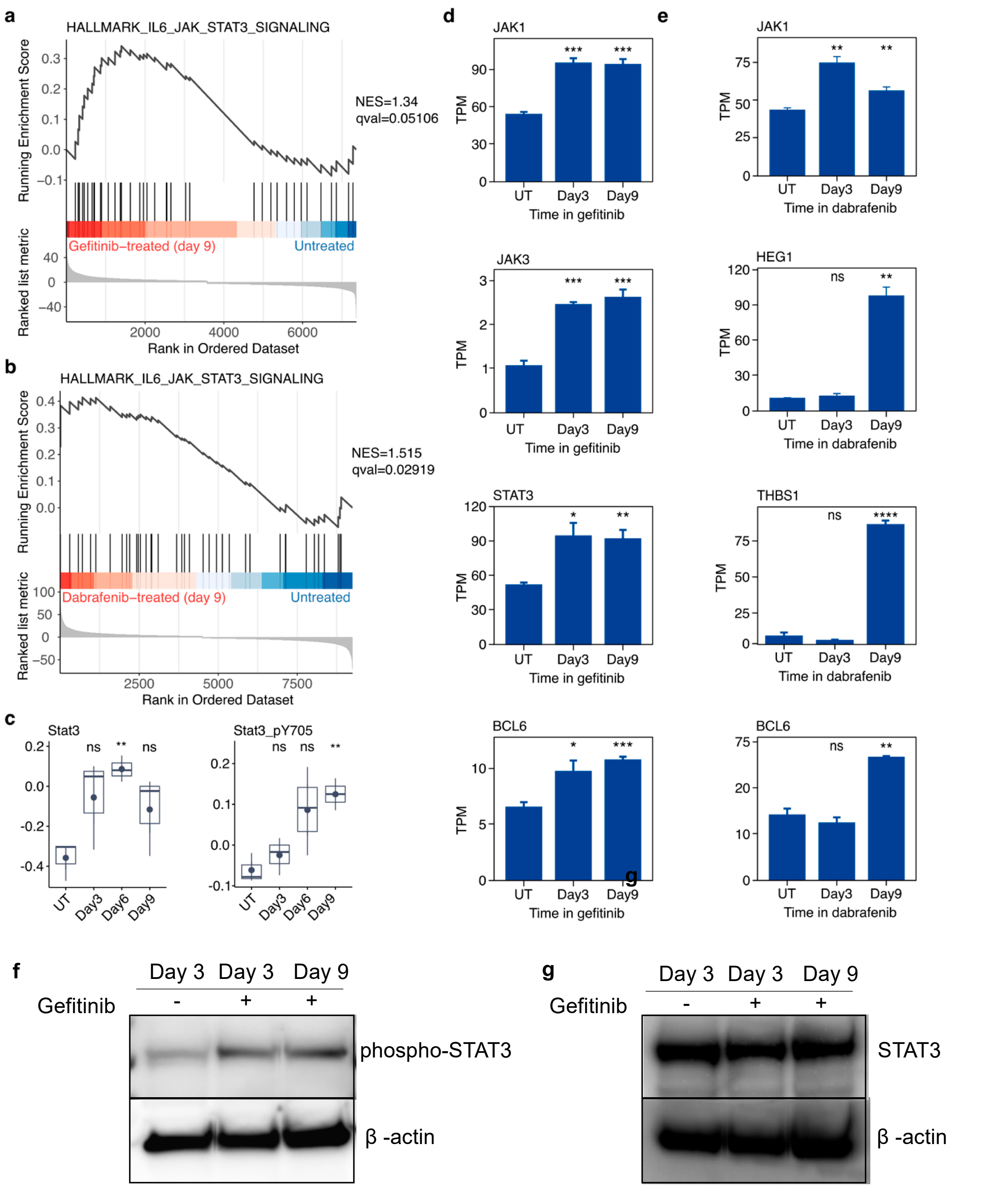

Figure S2. Gefitinib in PC9 cells and dabrafenib in SKMEL28 cells induces activation of the STAT3 signaling pathway in drug-tolerant cells. **a**) Hallmark gene set "IL6/JAK/STST3 Signaling" is enriched in PC9 cells treated with gefitinib. **b**) Hallmark gene set "IL6/JAK/STST3 Signaling" is significantly enriched in SKMEL28 cells treated with dabrafenib. **c**) RPPA quantification of proteins and phospho-proteins associated with STAT3 signaling, including STAT3, and phospho-STAT3 (Y705), from PC9 cells in

gefitinib for 3, 6, or 9 days along with untreated control cells (UT). **d,e**) Normalized transcript abundance levels of several genes associated with IGF signaling in PC9 cells in gefitinib (**d**) or SKMEL28 cells in dabrafenib (along with untreated control cells (UT).) for 3 or 9 days along with untreated control cells (UT). **f)** Western blot analysis of phospho-STAT3 (Tyr705) from untreated SKMEL28 and SKMEL38 cells in dabrafenib for 1 day or 3 days. **g)** Western blot analysis of total STAT3 protein from untreated SKMEL28 and SKMEL38 cells in dabrafenib for 1 day or 3 days. **h**) original, unprocessed version of the phospho-STAT3 Western blot **i**) original, unprocessed version of the total STAT3 Western blot. Data are represented as the mean ± SEM (n = 3). Two-tailed Student's t test was used for comparisons between two groups. *P < 0.05, **P < 0.01, ***P < 0.001, ****P < 0.0001, ns = P > 0.05.

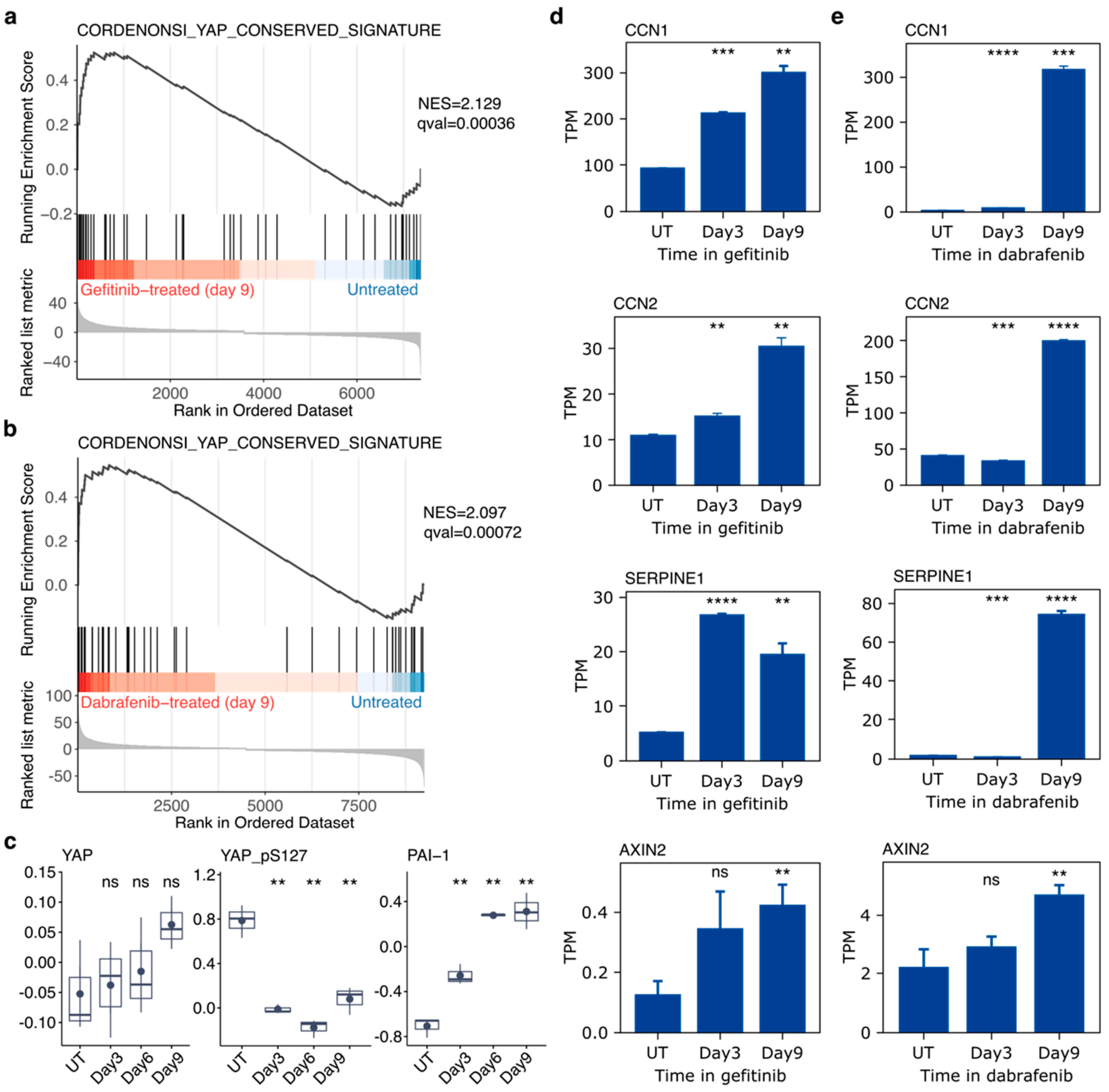

Figure S3. Gefitinib in PC9 cells and dabrafenib in SKMEL28 cells induces activation of the YAP pathway in drug-tolerant cells. **a**) GSEA NES scores indicate the gene set "YAP Conserved Signature" is significantly enriched in PC9 cells treated with gefitinib for 9 days, relative to untreated PC9 cells. **b**) GSEA NES scores indicate the gene set "YAP Conserved Signature" is significantly enriched in

SKMEL28 cells treated with dabrafenib for 9 days, relative to untreated SKMEL28 cells. **c**) RPPA quantification of proteins and phospho-proteins associated with YAP signaling, including YAP, phospho-YAP (S127), and PAI1, from PC9 cells in gefitinib for 3, 6, or 9 days along with untreated control cells (UT). **d,e**) Normalized transcript abundance levels of several genes associated with YAP signaling including CCN1, CCN2, SERPINE1, and AXIN2 in PC9 cells in gefitinib (**d**) or SKMEL28 cells in dabrafenib (**e**) for 3 or 9 days days along with untreated control cells (UT). Data are represented as the mean ± SEM (n = 3). Two-tailed Student's t test was used for comparisons between two groups . $^{*}P < 0.05$, $^{**}P < 0.01$, $^{***}P < 0.001$, $^{****}P < 0.0001$, ns = $P > 0.05$.

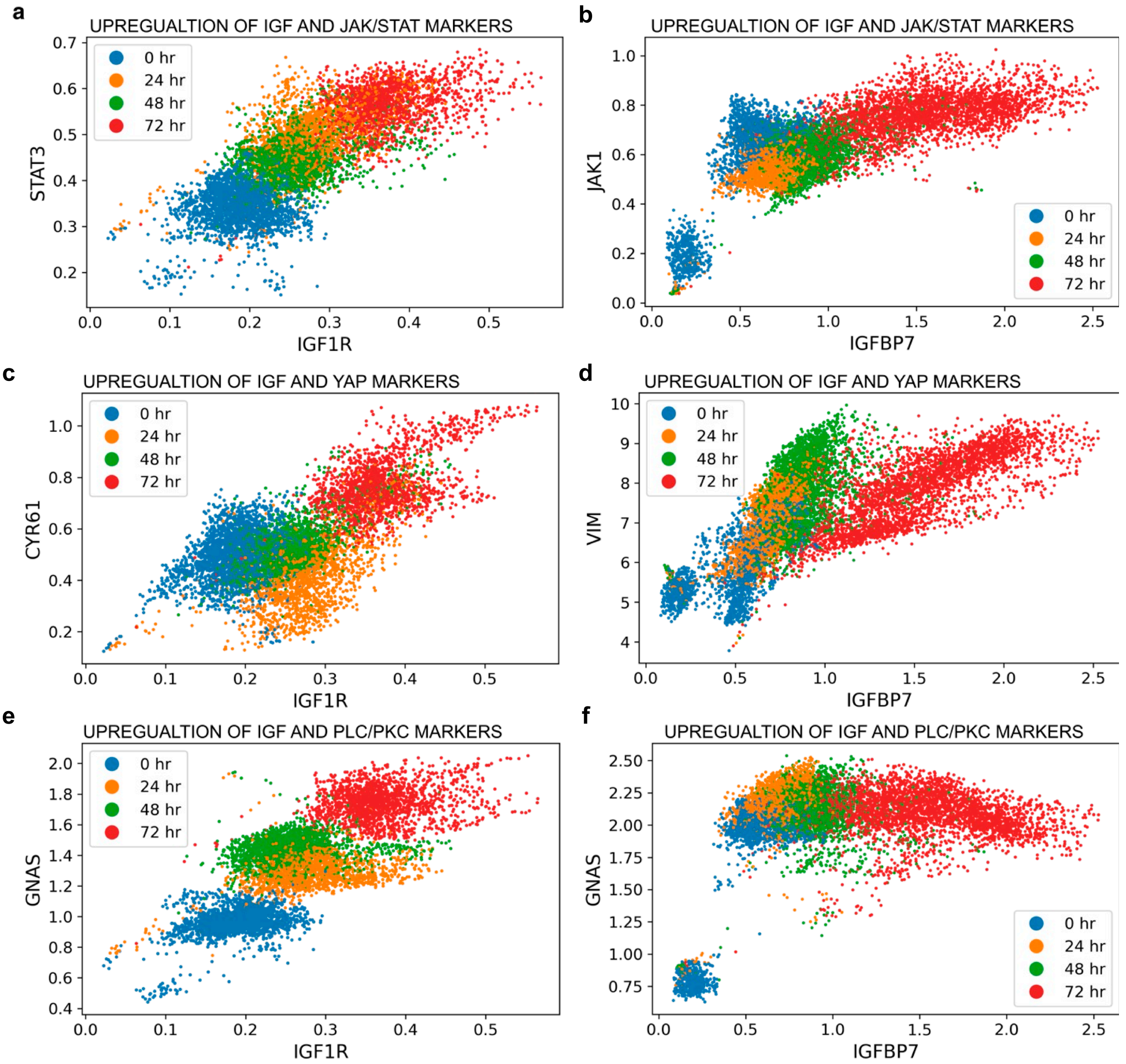

Figure S4. Upregulation of markers for alternate mitogenic signaling pathways during the development of drug tolerance. **a,b**) Markers of IGF signaling (IGF1R or IGFBP7) and JAK/STAT signaling (STAT3 or JAK1) are simultaneously upregulated in single PC9 cells treated with gefitinib (**a**) or single SKMEL28 cells treated with dabrafenib (**b**). **c,d**) Markers of IGF signaling (IGF1R or IGFBP7) and YAP signaling (CYR61 or VIM) are simultaneously

upregulated in single PC9 cells treated with gefitinib (**c**) or single SKMEL28 cells treated with dabrafenib (**d**). **e,f**) Markers of IGF signaling (IGF1R or IGFBP7) and PLC/PKC signaling (GNAS) are simultaneously upregulated in single PC9 cells treated with gefitinib (**e**) or single SKMEL28 cells treated with dabrafenib (**f**).

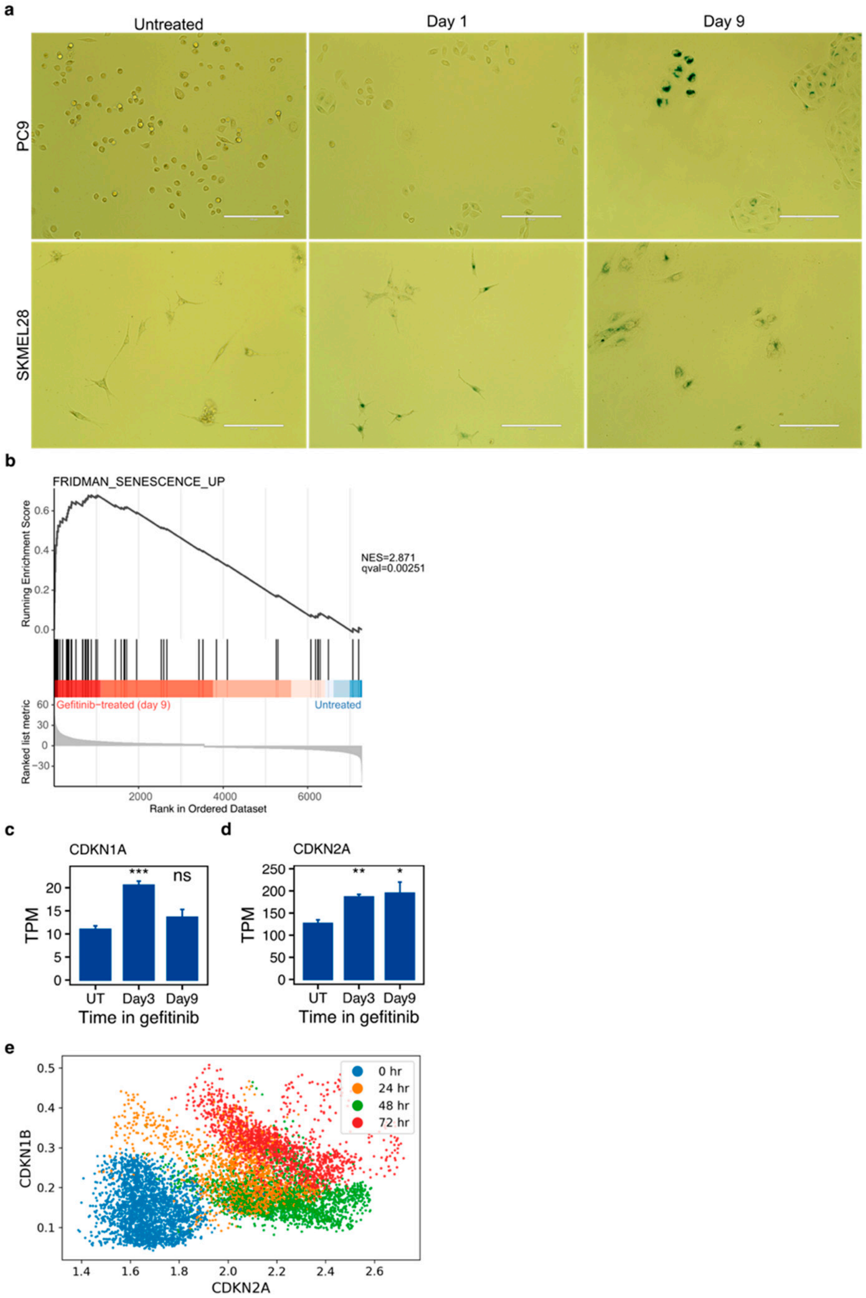

Figure S5. Features of cellular senescence accompany the emergence of drug tolerance. **a**) Brightfield images of PC9 cells (top) and SKMEL28 cells (bottom) cultured in targeted therapy for the indicated time and subsequently for stained SA-β-gal activity. The scale bar in each image corresponds to 200 μm. **b)** GSEA plot showing the relative enrichment of the “Fridman Senescence Up” gene set in gefitinib-treated PC9 cells. **c,d**) Normalized transcript abundance of CDKN1A (c) and CDKN2A (d) in PC9 cells treated

with gefitinib for 0, 3, or 9 days. **e)** Gene-gene relationship of CDKN2A and CDKN1B from MAGIC imputation analysis performed on scRNA-seq of PC9 cells in gefitinib for 0, 24, 48, or 72 hours. Data are represented as the mean ± SEM (n = 3). Two-tailed Student's t test was used for comparisons between two groups (untreated vs time point) for statistical analyses. *P < 0.05, **P < 0.01, ***P < 0.001, ****P < 0.0001, ns = P > 0.05.

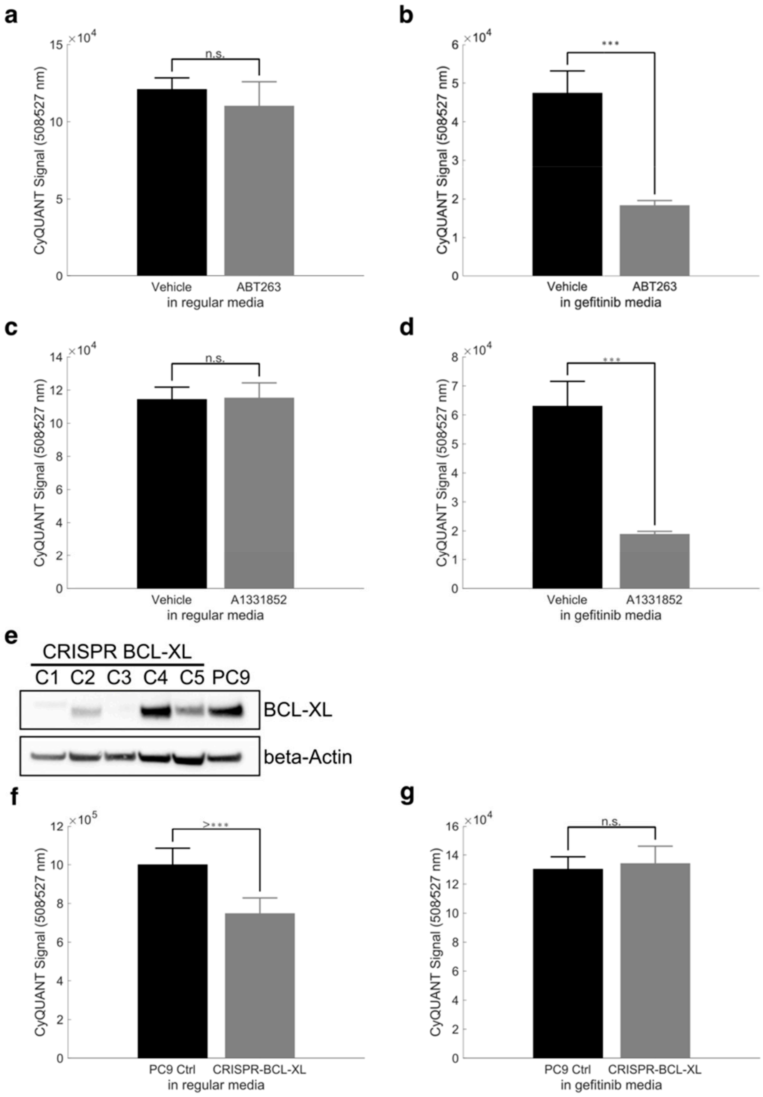

Figure S6. Pharmacologic but not genetic inhibition of BCL-XL results in increased gefitinib efficacy. **a,b)** Quantification of PC9 cell growth in normal media or gefitinib-containing media treated with vehicle (DMSO) or Navitoclax (ABT-263). **c,d**) Quantification of PC9 cell growth in normal media or gefitinib media treated with DMSO or A1331852. Data are represented as the mean ± SD (n = 3). Two-tailed Student's t test was used for comparisons between two groups. *P < 0.05, **P < 0.01, ***P < 0.001, ****P < 0.0001, ns = P > 0.05. **e)** Western blot analysis of BCL-XL protein from parental PC9 cells or clonal populations (C1-C5) of CRISPR-BCL-XL PC9 cells, with beta-actin serving as a loading control. **f,g**) Quantification of the growth of parental PC9 cells and CRISPR-BCL-XL PC9 cells (Clone 3) in normal media and gefitinib media. Data are represented as the mean ± SD (n = 8). Two-tailed Student's t test

was used for comparisons between two groups for statistical analyses. *P < 0.05, **P < 0.01, ***P < 0.001, ****P < 0.0001, ns = P > 0.05.

a

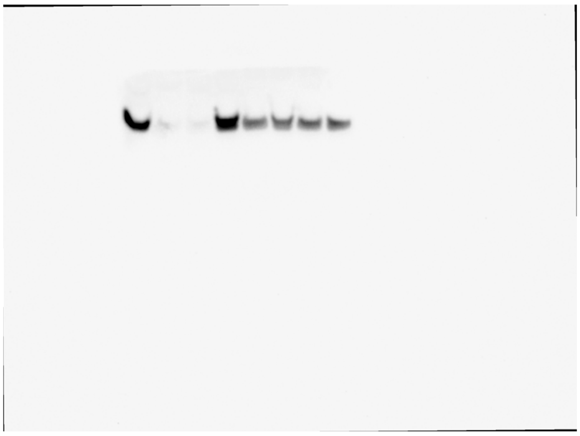

b

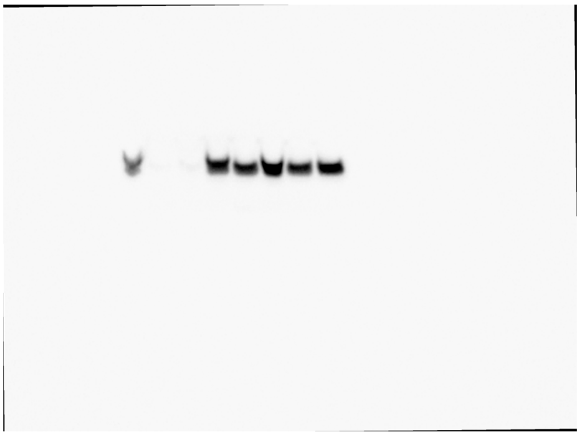

c

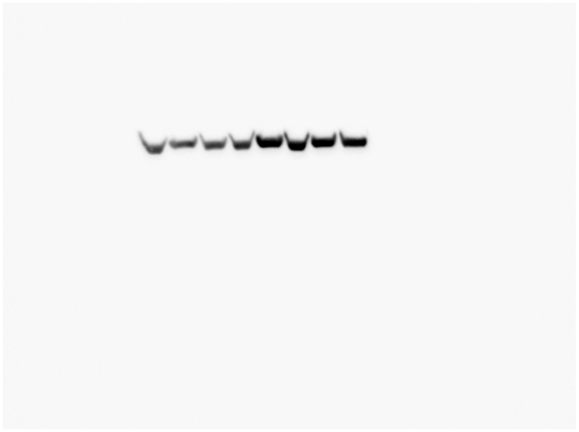

Figure S7. Original, uncropped images of Western blots from Figure 3. **a**, panel 3F, phospho-S6 (S240/244) **b**, panel 3F: phospho-S6 (S240/244) **c**, panel 3F, β-actin.

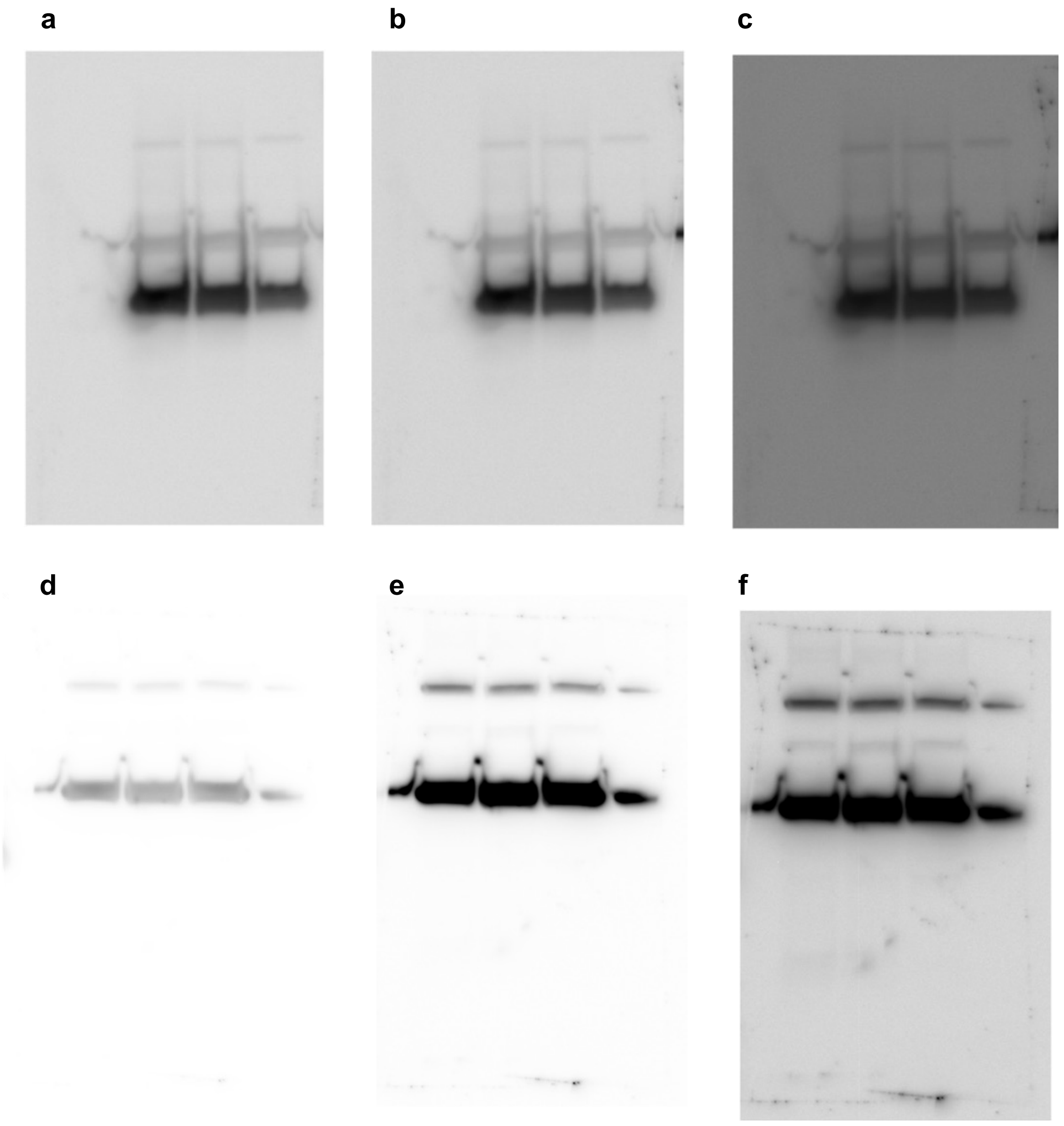

Figure S8. Original, uncropped images of Western blots from Figure 3**. a-c**, different exposures of the blot for panel 3G, total S6 protein **d-f**, different exposures of the blot for panel 3G, β-actin.

a

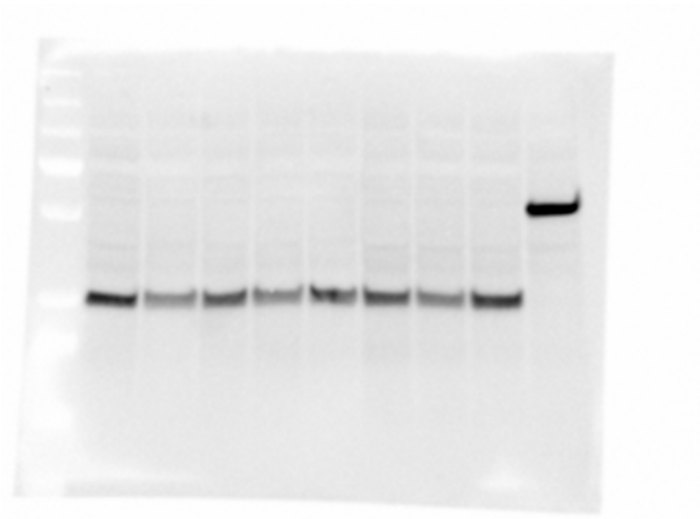

Figure S9. Original, uncropped images of the Western blots from Figure 7C**.**

a

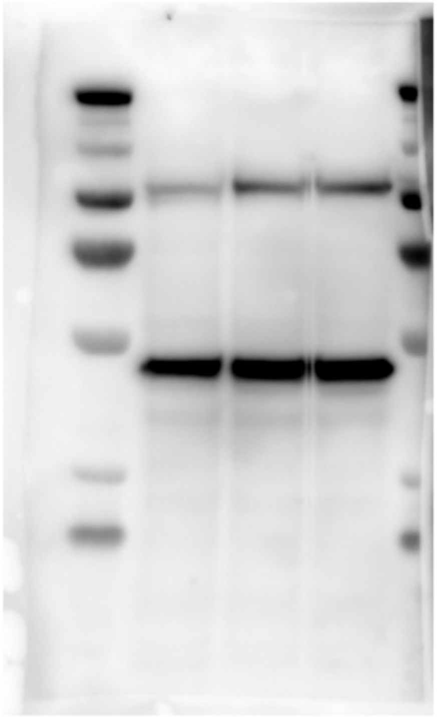

b

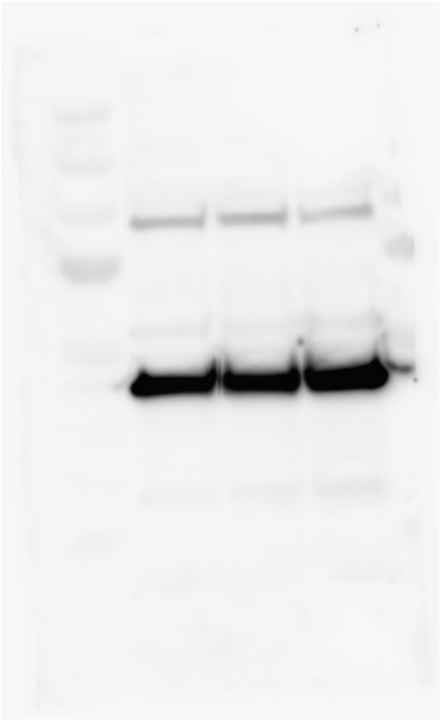

c

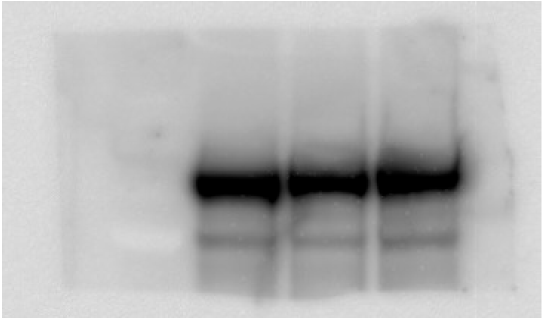

d

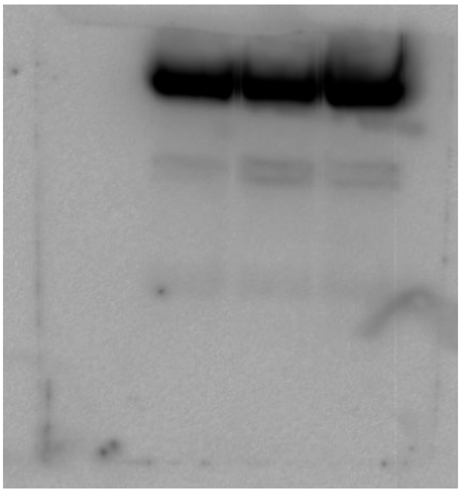

Figure S10. Original, uncropped images of Western blots from Figure S2. **a**, panel S2F, phospho-STAT3 **b**, panel S2F, β-actin **c**, panel S2G, STAT3 **d**, panel S2G, β-actin

a

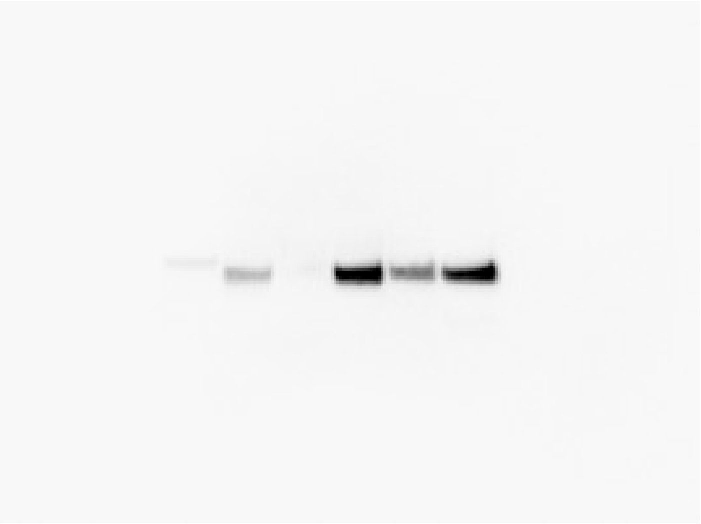

b

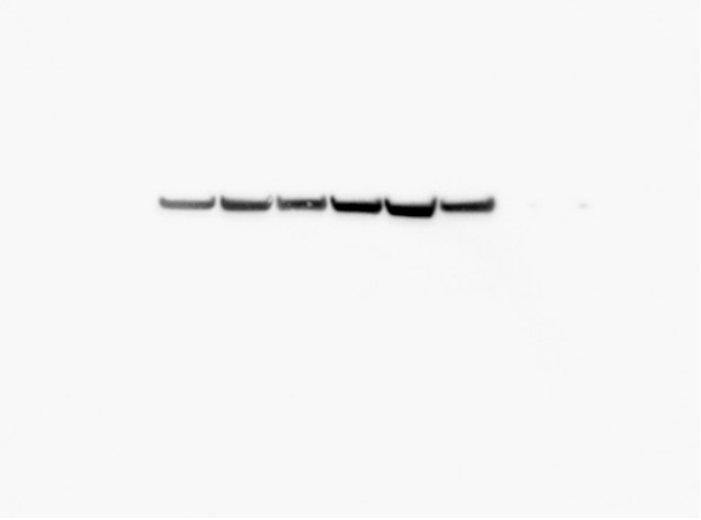

Figure S11. Original, uncropped images of Western blots from Figure S6. **a**, panel S6E, BCLXL **b**, panel S6E, β-actin
